# A Critique of Vanishing Voice in Noncooperative Spaces: The Perspective of an Aspirant Black Female Intellectual Activist

**DOI:** 10.1007/s10551-022-05111-3

**Published:** 2022-04-16

**Authors:** Penelope Muzanenhamo, Rashedur Chowdhury

**Affiliations:** 1 Michael Smurfit Graduate Business School, University College Dublin, Dublin, Ireland; 2 Southampton Business School, University of Southampton, Highfield, Southampton, SO17 1BJ UK

**Keywords:** Intellectual activism, Intersectionality, Racial equality

## Abstract

We adopt and extend the concept of ‘noncooperative space’ to analyze how (aspirant) black women intellectual activists attempt to sustain their efforts within settings that publicly endorse racial equality, while, in practice, the contexts remain deeply racist. Noncooperative spaces reflect institutional, organizational, and social environments portrayed by powerful white agents as conducive to anti-racism work and promoting racial equality but, indeed, constrain individuals who challenge racism. Our work, which is grounded in intersectionality, draws on an autoethnographic account of racially motivated domestic violence suffered by our lead author. Our analysis suggests that (aspirant) black women intellectual activists must develop courage to sustain their ‘voice’ within noncooperative spaces. However, the three interlinked dimensions of noncooperative spaces—namely, deceiving design, hegemonic actors’ indifference to racism, and (some assimilated gatekeepers’) false equivalence—may gradually erode a black female scholar’s courage. This forces her ‘voice’ to vanish temporarily, or even permanently. Courage is thus fragile and depletable. Yet, courage can be regenerated, resulting in regaining voice. Consequently, we propose courageous collective action by white allies and black and brown individuals who voluntarily and officially cooperate within and across various spaces to achieve racial equality.

## Introduction

Recently, numerous hegemonic actors across institutions, organizations, and other social contexts have started publicly condemning racism and expressing empathy for racialized bodies (Ansell, 2016; Logan, 2019). However, racism remains deeply entrenched within institutions (e.g., academia), organizations (e.g., firms), and society, and continues to mutate and adapt similar to a virus (Nkomo, 2020). The simultaneity of hegemonic anti-racism rhetoric and pervasive racism is particularly visible in academia (Boykin et al., 2020) where marginalized stakeholders (Derry, 2012), such as black female scholars, are persistently devalued (Dar et al., 2020). Marginalized stakeholders are individuals who lack self-representation, and they are ignored, neglected, mistreated, misrepresented through bias, and discriminated against “even when they make a meaningful social contribution” (Chowdhury, 2021a, p. 2).

Notwithstanding, a few black female scholars, particularly within management and organization studies (MOS), fundamentally lead in the struggle for racial equality (e.g., Bell et al., 2021; McCluney & Rabelo, 2019; Morgan Roberts & Mayo, 2019; Nkomo, 2020). Categorically, their attempts at combating racism reflect intellectual activism, which denotes “the myriad ways that people place the power of their ideas in service to social justice” (Collins, 2013, p. ix). Historically, black women thinkers are at the vanguard of exposing and challenging the ‘othering’ of black bodies as inferior to white persons (cf. Said, 1978), and its damaging effects upon individual, collective, and overall societal progress along socio-political and economic dimensions (e.g., McCluney & Rabelo, 2019; Proudford, 1999; Proudford & Thomas, 1999).

The earliest recorded intellectual activism among black female scholars is traced back to women such as Sojourner Truth, a nineteenth-century abolitionist, preacher and women’s rights activist, and Anna Julia Cooper whose black feminist scholarship fundamentally informs scholarship on intersectionality (Gilbert et al., 1991). However, achieving racial equality across time and space appears elusive even when increasing numbers of scholars from diverse social backgrounds are joining the struggle (e.g., Agyemang et al., 2020; Chowdhury, 2021b; Logan, 2019; Netto et al., 2020; Ozturk & Berber, 2022; Wang & Seifert, 2020). We emphasize the limited progress towards eliminating racism in ‘noncooperative spaces’ (Chowdhury, 2021c). Borrowing from Chowdhury’s (2021c) conception, we represent noncooperative spaces as institutional, organizational and social environments (e.g., domestic contexts) designed and portrayed by powerful white agents to appear, on the surface, supportive to intellectual activism, dedicated to racial equality, and thus victim-friendly and protective. Yet such settings are patronizing and dangerous for individuals who (dare to) challenge racism.

In using the concept of ‘noncooperative spaces’ we aim to explore how (aspirant) black female intellectual activists attempt to sustain their efforts through ‘voice’, and how they cope within seemingly welcoming and accommodating institutional, organizational and social contexts that are, however, intensely hostile to the individuals. We consider voice as speaking-up behavior proactively exhibited by employees when they suggest ways for achieving change (van Dyne et al., 2003). Our work is grounded in intersectionality (Crenshaw, 1989, 1991), and it draws on an autoethnographic account (Ellis et al., 2011) of our lead author, the only black (African) female scholar at a European Business School (EBS), who suffered racially motivated domestic violence. Our study contributes to the body of (Black) scholarship with a predominant focus on intellectual activism and racial equality.

This research establishes that (aspirant) black female intellectual activists must develop courage to sustain ‘voice’ within noncooperative spaces. However, noncooperative spaces tend to temporarily *empower* and, at the same time, continuously *disempower* a black female scholar through three interwoven dimensions. We identify these dimensions as deceiving design, powerful white actors’ indifference to racism, and (some assimilated gatekeepers’) false equivalence. These dimensions gradually erode the courage of a black female scholar, which forces ‘voice’ to vanish temporarily or even permanently (Woodyard & Gadson, 2018), thus disrupting intellectual activism. Courage, which is often ascribed to managerial (Sekerka et al., 2009) and leadership areas (Solinger et al., 2020) is fragile and nebulous at best. Courage fades and regenerates, resulting in (re)gaining voice. We further observe that courage is neither a (white) manly attribute as traditional discourse suggests (Jablin, 2006; Rate & Sternberg, 2007), nor is it ‘somewhat stable’ (Hannah et al., 2011).

To sustain commitment to truly achieving racial equality and embedding its broader impact, we suggest *courageous collective action* among black (and brown) individuals and white allies. This potentially allows the actors to support one another and *cooperate across institutional/organizational and social contexts* to achieve a more equitable society. Our analysis starts by reviewing studies on intersectionality and ‘noncooperative spaces’, before presenting and analyzing our autoethnographic materials. We conclude with an articulation of agential and organizational/institutional implications.

## Theoretical Context

### Intersectionality

Researchers apply intersectionality in multiple and often inconsistent ways (Jordan-Zachery, 2007) to explore the experiences of marginalized global communities (Collins, 2015; Collins & Bilge, 2020) which include Asian cis-heterosexual male leaders (Liu, 2019b), gay and queer individuals (Rahman, 2010), and niche scientists (Styhre, 2018). However, our view of intersectionality is consistent with Crenshaw’s (1991, p. 1244) conception as “the various ways in which race and gender interact to shape the multiple dimensions of Black women's employment [and domestic violence] ‘experiences’”. The social location of black women at the intersection of not only gender, race, class, and ethnicity but also ability, nationality (Yuval-Davis, 2006), and according to stereotypes (Johnson-Ahorlu, 2012; Reynolds-Dobbs et al., 2008) means that they suffer unique experiences of violence—racism and subjugation—within institutions, organizations, and society (Collins & Bilge, 2020).

Broadly observed, black individuals, women and Two-Spirit, Lesbians, Gays, Bisexuals, Transgenders and Queers or Questioning (2SLGBTQ+s) encounter discrimination in social and professional settings. Notwithstanding, all women or all black people are *not* the same, implying that black women’s experiences are not adequately captured by either of these broad social categories (Crenshaw, 1989, 1991). For example, compared to black women, white women structurally benefit from white privilege, defined as better life chances and outcomes for all white individuals due to their race, regardless of the state of their life conditions (Taylor Phillips & Lowery, 2015). This means that, structurally, white women have better access to opportunities, resources, and loci of power (white males within institutions and organizations) than black women (and black men) have. Furthermore, social hierarchies position black women below black men and white women, rendering them the first group to be eliminated from institutions and organizations in times of economic hardship (Crenshaw, 1989, 1991).

In addition, black women’s lived experiences of subordination and subjugation must be understood within the specific contexts where they are produced (Jordan-Zachery, 2007) in order to expose how a particular socially constructed dimension implicates others jointly responsible for the structurally powerless position of a black woman within a given location. This deserves a study of the experiences of black women scholars situated within business schools linked to the attainment of racial equality (Dar et al., 2020; Muzanenhamo & Chowdhury, 2022).

### Noncooperative Spaces

For many female black scholars working towards achieving racial equality, speaking up against racism often feels as if they are “banging” their heads against a “brick wall”: “The wall keeps its place”, therefore it is only the individuals whose heads get sore (Ahmed, 2012, p. 156). Ahmed’s (2012) metaphorical analogy highlights how intellectual activism more often than not achieves minimal impact, if any, granted that black female scholars’ “antiracist, anti-sexist” and “postcolonial” voices (Mirza, 2009, p. 2) inevitably evolve within ‘noncooperative spaces’ (Chowdhury, 2021c). The latter, ‘noncooperative spaces’, allow black female scholars’ voices to be developed and articulated (Cornelius et al., 2010) while simultaneously blocking the voices from achieving any significantly transformative results.

In this paper, we interpret noncooperative spaces based on Chowdhury's (2021c) conception in relation to marginalized stakeholders’ entrepreneurial capacities for thriving and leading a dignified life within a refugee environment. More explicitly, we define and extend Chowdhury’s (2021c, p. 4) notion of ‘noncooperative spaces’ as “highly restrictive, disadvantageous, or even harmful [institutional, organizational and social environments] because of institutional arrangements” that inhibit racialized individuals’ voice and capacity to obtain justice and/or co-transform racist structures with white actors who seek a more equitable society. Eliminating racism involves a joint effort between white and non-white bodies, and is thus unattainable without either of these two broad categories’ input (Bell et al., 2021; Contu, 2020; Edmondson et al., 2020; Swan, 2017).

Based on our analysis and integration of literature on the documented racialized experiences of black (and brown) scholars (Ahmed, 2021; Muzanenhamo & Chowdhury, 2021; Nkomo, 2016), and diverse forms of (in)equalities and (business) ethics, we suggest the following as the interlinked dimensions of noncooperative spaces: (i) a deceiving design (Ahmed, 2012, 2021; Chelliah & Swamy, 2018; de Vries et al., 2012; Jehn & Scott, 2008; Olekalns & Smith, 2007); (ii) indifference (Acker, 2006; Frankenberg, 1993; Heffernan, 2011; Latané & Darley, 1970); and (iii) false equivalence (Baron & Jost, 2019; Cooper, 2010; Springer & Özdemir, 2022).

*First*, noncooperative spaces can deceive black female intellectual activists into perceiving (some) powerful white actors’ proclamations feigning support for racial equality as genuine, by concealing and omitting details, or disseminating false information on what the agents truly think and how they feel about racism, as well as their actual intentions regarding tackling the issue (Ahmed, 2021; Chelliah & Swamy, 2018; Jehn & Scott, 2008). Contemporary settings in which anti-racism rhetoric is produced are thus *not overtly racist*, as hegemonic actors may publicly, and in theory, endorse policies and initiatives targeting racial equality (Ballard et al., 2020). Such statements, slogans and campaigns are, however, to all intents and purposes, empty promises (Ballard et al., 2020; Boykin et al., 2020).

Furthermore, powerful white actors *rarely (directly) articulate the ways* in which voices seeking racial equality are to be suppressed and subjugated (cf. Olekalns & Smith, 2007). Rather, suppressive practices are implied in the agents’ penalization of efforts targeting racial equality by employing rationalized discourses that hide racism (Boykin et al., 2020; Chelliah & Swamy, 2018; Jehn & Scott, 2008). Primarily hegemonic articulations of racial equality do not transform the power structures that privilege white individuals (Nkomo & Al Ariss, 2014); nor do they truly permit racialized bodies and anyone who participates in debates on racial equality to live a dignified life (Chowdhury, 2021a, b). Thus, noncooperative spaces misleadingly give hope to black scholars that their voices *are* being heard by powerful white actors and that change *will* materialize (Liu, 2019a; also see de Vries et al., 2012).

*Second*, suppression and subjugation are further reflected in the hegemonic actors’ and *assimilated gatekeepers’ indifference* to racism. By assimilated gatekeepers we mean traditionally marginalized non-black women empowered and integrated into the hegemonic structures by powerful white male actors (Fotaki, 2013; Horn, 1997). Indifference signifies complete lack of concern and care for, or empathy with the racialized individuals, to the extent that assimilated non-black women ignore racism (Acker, 2006; Frankenberg, 1993; Heffernan, 2011; Latané & Darley, 1970). While such assimilated actors are cognizant of structural inequalities, their quest for legitimacy and acceptance by hegemonic actors prompts them to avoid challenging the status quo (Fotaki, 2013; Herman et al., 2013; Horn, 1997).

Hence, assimilated gatekeepers may effectively interpret, rationalize and defend racist practices underpinning noncooperative spaces as preserving the standards of professional excellence (Cox, 2004), resulting in inaction against racism (Boykin et al., 2020). Furthermore, such agents potentially intimidate (aspirant) black (and brown) female intellectual activists, as exemplified by Liu and Pechenkina's (2016) study on assimilated gatekeepers’ tolerance of powerful white actors’ display of visual signage reinforcing racism within an academic institution. Beyond this, hegemonic actors’ internal ridiculing of the fight for equality, while donating to movements such as Black Lives Matter (and 2SLGBTQ+s causes) to boost corporate image (Ahmed, 2012), represents another symbolically racist practice underpinning noncooperative spaces.

*Third*, both a deceiving design and indifference to racism can trigger false equivalence, particularly among assimilated gatekeepers and ‘liberal’ white individuals (Wright et al., 2007). False equivalence reflects a form of flawed reasoning and rhetoric that erases distinctions between two somewhat related phenomenon, and gives equal weight to each (Baron & Jost, 2019; Cooper, 2010; Springer & Özdemir, 2022). Meghji and Saini (2018) note that false equivalence presumes that all voices and experiences pertaining to an issue are equal, have equal significance, and must be equally accommodated.

For instance, non-black actors may regard the experiences associated with gender-based discrimination and racism as similar, when they are indeed distinct (Crenshaw, 1989, 1991). Therefore, a myopic tendency among some white female individuals in particular leads them to generalize their experiences as representative of all women, and consequently, their failure to demolish noncooperative spaces and tackle racism. However, scholars, particularly female black feminists, challenge this inclination as it fails to consider white privilege (Collins, 2002; Davis, 2011), and mobilize action towards dismantling noncooperative spaces. Furthermore, ‘liberal’ white actors tend to be empowered ‘color-blind’ individuals who hold the conviction that skin color (or ‘race’) is immaterial and that all humans are equal (Wright et al., 2007). Consequently, such liberal agents are inclined to evoke notions of meritocracy, while neither challenging the status quo nor implementing any impactful actions to support black female intellectual activists and combat racism.

To maintain the status quo (Grimes, 2001), scholars (Ahmed, 2012, 2021) suggest that noncooperative spaces subtly—yet insidiously through their dimensions—induce anxiety and fear of reprisals, demotions and job losses, not only in subjugated individuals overtly challenging racism (e.g., Dar, 2019; Harlow, 2003; Settles et al., 2019), but also among white individuals who might otherwise speak out (i.e., articulate voice) against marginalization (Ashburn-Nardo et al., 2008; Meyerson & Scully, 1995). Fear is a “hidden, controlled, and privately lived” (Haas, 1977, p. 156) anticipation of sanctions from powerful actors for violating their rules or deviating from their prescribed behavioral standards (Higgins, 1987).

The various undesirable outcomes of expressing ‘voice’ against racism may translate into individuals’ pessimistic assessment of the risk associated with such action and a heightened sense of being controlled by the situation (Lerner & Keltner, 2001). Subsequently, the actors may avoid speaking out, or withdraw their voice (DeCelles et al., 2020) from the pursuit of racial equality. Nonetheless, silence may protect the self from punitive consequences (van Dyne et al., 2003) while maintaining the status quo.

Within academia, hegemonic actors increasingly deploy racial equality discourses (Ballard et al., 2020). However, the academic space is historically racist (Wilder, 2013) and sexist (Nkomo, 1992). Thus, an investigation of how black female scholars—as marginalized stakeholders (Derry, 2012)—cope within noncooperative spaces, while performing and sustaining their intellectual activism is long overdue. We subsequently address this by exploring a budding black African female intellectual activist’s experiences.

## Context and Methodology

To understand how (aspirant) black female intellectual activists potentially navigate noncooperative spaces, we trace the journey of one of our co-authors, Alice (alias). Alice is a black African female scholar from Amare (pseudo name for her home country), educated in Europe where she lived, and worked on a temporary contract at a EBS in EC (pseudo country name). As an adopted child of a white European father, with the experience of living in both Africa and Europe, Alice developed a passion for racial equality in academia during her early college years. Her passion was fueled by observing MOS’ inclination to forget, ignore, and only partially represent black people and African business contexts (Nkomo, 2016). Alice subsequently pursued doctoral research in her area of interest, and landed a job connected to her passion soon after graduation. She started documenting her work-related experiences as soon as she was recruited.

Our research draws on Alice’s autoethnographic account. Autoethnography represents an approach to conducting and writing research with the goal of describing and systematically analyzing personal experiences and their linkage to the broader socio-cultural context (Ellis et al., 2011). An autoethnographic inquiry involves exposing the researcher’s vulnerable self, body and emotions and the production of evocative stories that allow readers to develop compassion and empathy. Autoethnography presents intimate details and concrete meaningful experiences that potentially facilitate readers’ understanding of “how to live and cope” (Ellis, 1999, p. 669). Researchers have drawn critical attention to the methodology for its perceived degree of subjectivity (Denzin, 2006), narcist orientation, and poor rigor (Doloriert & Sambrook, 2012; Learmonth & Humphreys, 2011). Nonethless, in essence, autoethnography challenges the canonical approaches to research that are predicated on the dominant perspective of the white heterosexual and a middle- or an upper-class robust man (Ellis et al., 2011).

Non-white female scholars frequently adopt autoethnography to interrogate racist experiences and deploy intellectual activism as an attempt to achieve racial equality (Bell & Nkomo, 1999; Hernandez et al., 2015; Liu & Pechenkina, 2016). Similar to such scholars, Alice’s personal experience is situated within the broader social, political and economic experiences of black (female) bodies working in Western-centric academia. Alice’s narrative focuses on specific vignettes and Epiphanes that define her role as an aspirant intellectual activist within a Western-centric environment. Her vignettes are presented retrospectively and selectively (Denzin, 2006; Ellis et al., 2011).

By co-constructing and co-producing this autoethnography consistent with similar studies (Bourgoin et al., 2020; Fernando et al., 2019), Alice produced the vignettes herself based on memory and supported with her journal entries (Liu, 2019a). Memory use is conventional to most qualitative research granted that investigators depend on interviewees’ recollections of and “best attempt[s]” to reproduce “what is (or was) going on here – or there”  (Davies & Gannon, 2006, p. 1). Thus, consistent with other autoethnographic accounts, we strive to offer a comprehensive narrative (Ellis et al., 2011; Fernando et al., 2019; Liu & Pechenkina, 2016). For this our second author listened to Alice’s narrative, reflected on it, questioned it, and read multiple drafts re-written and refined by Alice across a 3-month period (Basner et al., 2018; De Schauwer et al., 2018).

While our interpretation of Alice’s vignettes is grounded in our collective understanding of her narrative, the development of the analysis is led by our second author with Alice’s contributions (Basner et al., 2018; Bourgoin et al., 2020; Fernando et al., 2019). The second author has expertise on ‘noncooperative spaces’, marginalized stakeholders, and ethics, which lends an analytical dimension to our evocative account. Critically reflecting on Alice’s vignettes supports the development of a socially situated reconceptualization significant to the broader context of black scholars, intellectual activism, and racial equality (Fernando et al., 2019; Learmonth & Humphreys, 2011; Liu & Pechenkina, 2016).

Furthermore, contrary to some scholarly approaches that integrate vignettes with the analysis in the same section (van de Berg, 2021; Liu, 2019a), we adopt a story-telling approach that allows the reader to potentially immerse themself in our narrative, prior to our interpretation of all the vignettes (Boje & Tyler, 2009; Liu & Pechenkina, 2016). In addition, rather than retaining the lead author’s identity both in the narrative and its analysis (Fernando et al., 2019), we use the alias, Alice, to better demarcate the lead author’s joint roles as investigator and unit of analysis. This permits us to offer a more depersonalized and critical analysis of relevance beyond a single individual (Bourgoin et al., 2020). We have taken relevant anonymization measures to ensure the safety (van de Berg, 2021) and ethical portrayal of others implicated in our study (Ellis et al., 2011; Fernando et al., 2019).

## Vignettes

### Emerging Scholar

*As the only black female scholar in the Business School and, to the best of my knowledge, possibly across all business schools in the country, I initially struggled to make any meaningful progress. I could not find anyone available for collaboration on research centered on black people, African contexts and racial equality within the school or country where the school is based. However, about a year into the job, I reached out to Shoikat (alias), a brown colleague in another EU country, whose research partially overlapped with my own. We initiated several projects, resulting in some success, and attracted academic recognition for our research on racial equality. I was subsequently invited to participate on international panel discussions on racial equality and in other collaborative research projects. I was delighted that I was joining the community of other black academics actively working to achieve black people’s equal representation specifically in business education and research.*

*I started believing that I was at a stage where my voice was being heard on a global level, also by powerful academic actors. I felt confident that I was emerging as a potentially influential intellectual activist. Furthermore, some of my anti-racism social media posts, including a short poem I had written, attracted thousands of likes and shares. I was thrilled that my black African voice was being heard. Finally, as folks colloquially say, I had achieved my so-called ‘15 minutes of fame’, and I was inspired.*

### White Power and Disruption

*My life changed shortly after getting some recognition for the research with Shoikat. I experienced a racially based assault, classified by police in EC as domestic violence. I was assaulted by Lina (pseudo name), a white European woman who moved into my rented two-bedroom apartment in the middle of the COVID-19 pandemic to replace a close friend who had left for employment-related reasons. Lina worked as some sort of analyst for a blue-chip company in EC metropolis. We both worked from home at the time.*

*Tensions started building up when I shared the news with Lina about my recognition for anti-racism research. Lina dismissed my research by equating racism to nepotism that was rampant in her home country. She claimed that many people in her country, especially women, could not get jobs unless they were related to company bosses and politicians. So, to her way of thinking, whether the issue was racism or not, the suffering was the same. There were no reasons why black people should consider themselves so awfully disadvantaged.*

*The mood in the apartment changed following that disagreement. Lina continuously made jokes about black people being too sensitive, yet their experiences are just similar to how white women suffer and this knowledge is common sense. She joked about how Black Lives Matter (BML) was not seen as an issue of concern in the company employing her. The company simply donated some money to a number of organizations supporting the movement to keep investors happy. She frequently joked about how people from ‘shitty’ countries were lucky to make it in EC metropolis, and how she had never talked to any black person before relocating to EC.*

*A few days after the argument, Lina ordered me not to access the kitchen or my private shower after 10.30 pm. However, I stood my ground on utilizing both facilities. Then a few days later, Lina hit me when I was returning from the kitchen where I had made a cup of tea. It was in fact way before 10.30 pm when I came back from the kitchen and saw her standing in front of my door blocking the way into my room. Suddenly she aggressively turned, shoulder-bumped me, slapped and shoved me several times, spilling tea everywhere. It took me a moment to realize that I was being intentionally assaulted. I was shocked.*

*I did not hit her back but I told her to stop, and that I would call the police if she continued to hit me. My words did not have an immediate impact. When she finally stopped hitting me and shoving me into my room, I took a video clip and some photos of the spilt tea, still in disbelief and denial at what had just happened to me. I then sat down contemplating whether or not to call the police as I doubted that they would support me. However, about 5 or 10 minutes later when I stopped shaking, I decided to contact the police, who arrived about 40 minutes later after my second call to them. When the police left, I set there on my bed with tears streaming down my face.*

*I could not understand that I had been physically assaulted. Legally as co-tenants, Lina and I had the same privileges and obligations. Thus, I asked myself several times if Lina would have slashed my privileges within the ‘equally-shared’ apartment, if I had been a white female academic that she had found in the premises and who was attracting global attention for her work. I felt that Lina wanted to exercise white dominance over me. I was aware that she was not comfortable with the fact that my academic achievements and financial circumstances were far better than hers. Based on her comments about my holding of several degrees and coming from Africa, I suspected that Lina was envious, and she did not see me as deserving of those accomplishments. I concluded that her sense of envy, and perhaps inferiority, and a desire to exercise white power over an African black female lecturer made her physically assault me.*

### Let Down

*Two days after the physical assault I moved out of the shared apartment into a hotel, while negotiating with the landlord through their estate agency to ensure my safety. Legally, tenancy safety was a responsibility for the landlord. I needed a quiet and warm place with internet access as I was teaching online. It was in the middle of a teaching semester and the COVID-19 pandemic had reached its peak. However, the landlord did not provide any assistance as, presumably, there was nothing they could do, and I could not end my tenancy unless the police had issued a report of the assault. At the same time, I could not get the police to issue a report, as they dismissed my case as minor and less of a priority.*

*As staying in hotels in EC metropolis was extremely expensive, I made arrangements to move to a nearby EU country, where a close friend initially took me in for about a week. Shortly after, I started moving from one (affordable) hotel to another, so that I could continue teaching. Hotels in that neighboring EU country were mostly empty due to the COVID-19 pandemic.*

*Although I immediately informed the police about the physical assault, they appeared completely disinterested and uncooperative. No legal action was taken to hold the abuser accountable. The first strange thing was that it took two phone calls and almost 40 minutes to get the police to come over to the apartment on the night of the assault. The second issue was that the two young white female officers who came did not take any statement that night and, based on what I could hear, they seemed to have a very cordial chat with Lina when they spoke to her. They promised to return the next day for my statement, which they did after I had made several follow-up calls. The police also made it very clear to me that taking the case further to court was not worthwhile, as there was ‘no visible physical damage’ and it was a ‘minor assault’.*

*A female police officer promised to call me the next day, but to date, has not got in touch. I made several follow-up calls to the responsible police station while abroad trying to speak to the female officer, but I never reached her. I also emailed both the female police officer and her superintendent but there has been no response regarding the progress of the investigation. The last time I called, I spoke to another female police officer who made me understand that my case was further down on the list and it would take many months to investigate. Thus, it did not matter how many times I would call the police. Consequently, I stopped calling, and to date, I have not heard anything from them.*

### Falling Star

*For almost a month after the assault I struggled to process the abuse. I struggled to understand how something like that could happen to me, and with the fact that I did not have a safe and stable place from where to teach, apart from a hotel room. I had nightmares and I woke up in the middle of the night drenched in sweat. It was hard to maintain mental balance. I did not have the strength to tell anyone about the physical assault and my circumstances then, apart from the close friend who helped me initially. I worried mostly about ‘my’ students, who insisted on seeing me live on camera during lectures. I was worried that they would notice the different hotel room walls and wonder what was going on. So, I decided to provide them with some very sketchy details about temporarily leaving my apartment and adaptation. I nervously joked about being somewhat ‘homeless’ and considered an essential worker, and therefore allowed to stay abroad in relatively ‘cheap’ hotel rooms.*

*Although I am very close to my adoptive father and my entire ‘white family’ I chose not to tell them about the physical assault. The assault was not something consistent with who I was, what I stood for, and how I had always interacted with white people. The assault was particularly not aligned with my ‘white family’s’ perception of the sort of trouble I could be involved with. The assault was also not consistent with the achievements I had made and the current reputation I seemed to be garnering as an anti-racism scholar. Somehow, I felt both too angry and humiliated to discuss the issue with my ‘white family’ or anyone else.*

*Informing EBS (my employer) about the physical assault and temporary ‘homelessness’ was equally dreadful for me. I feared drawing unnecessary attention as a black African female lecturer. I dreaded being seen as someone causing trouble in the middle of the pandemic and right at the point when George Floyd’s murder was still very fresh in people’s minds. Even though I knew that EBS might find out somehow since I had reported the physical assault to the police, I still chose to be silent.*

*Moreover, the assault coincided with my period of ‘temporary’ fame as a potential intellectual activist, which made me fear that I would raise suspicions over intentionally provoking or even inventing the physical assault to capitalize on it. I feared being seen as playing the race card to retain a presence in the limelight. Experience taught me that, we, black people, are always regarded with suspicion. There is always a way to blame us for racism (we do it to ourselves) despite growing institutional rhetoric in support of equality. Most powerful white people do not actually care about racism as long as the issue does not generate negative publicity for their organizations.*

*Subsequently, I withdrew completely from social media or any such platforms that would have made me visible as an aspirant intellectual activist. I abstained from joining any discussions about racism and, for quite a while, I think I stopped existing outside hotel rooms and online lectures. Slowly, I started convincing myself that calling out racism was not really a priority for me. I was just a nobody who should work hard to ‘deserve’ and keep her job.*

### Re-emerging

*I started reflecting on everything that I and many more black people had been through during the previous year. It had been a tumultuous year, with COVID-19 disproportionately killing more ethnic minorities than other demographics. Racism was increasing. In the US, the police had recently killed George Floyd. I thought about the BLM movement and pressure on organizations to publicly admit their failure to eliminate racism. I reflected on this whole journey of intellectual activism; what was said, transformed, ignored, and maintained; by who, when and where; and the role I had played so far. In general, more non-white scholars and white allies were speaking out against racism. I told myself to disclose the physical assault, but I was scared, and in too much pain to do so.*

*Neverthless, I recounted some black female scholars’ experiences in the struggle for racial equality and their accomplishments not just for black bodies, but also for other marginalized voices. Those black female academics had risked a lot by ‘speaking truth to power’ and ‘telling truth to the people’ as Patricia Hill Collins inspired intellectual activists to do. I particularly appreciated that my globally recognized research had only achieved that status because my predecessors broke the ice and scaled the wall or took a seat on the bus. They were (and are) courageous black female scholars who risked losing their (professional) existence by speaking out against racism. I saw courage in their words and deeds. Then I decided to be brave and courageous, and told myself to speak out somewhere and somehow, even if that meant an uncertain future. That future was for me to embrace, in the same way that other black female academics resisted white power and, in so doing, paved the path, although rocky, for others like me.*

*I also told myself that it was my responsibility to speak out professionally against any form of racism affecting black bodies in work and domestic contexts. It was my obligation to resume my journey as a potential intellectual activist, just like other black scholars had made it their vocation to speak out on behalf of other black people. I did not know how many black or African women were beaten up (both physically and verbally) by some white women who sought to exercise white power. But I believed that my experience was not unique. Therefore, if I could not speak out on social media or through the police and the landlord, and to my employer or to blue-chip companies mocking BLM, I needed to explore other platforms.*

*Not speaking out was not only cowardly but, also, it meant that I condoned the racist physical assault. My silence also meant that I was in agreement with the indifference shown by the police, the housing agent, and a business education system that ignored teaching racial equality to its students. Furthermore, my silence potentially contributed to the perpetuation of such suffering among black women academics at the hands of white women professionals like Lina who physically assaulted such bodies in their (supposedly) ‘safe’ spaces.*

*I became convinced that if any other black female scholars faced white power in the form of physical assault by some white women, and they hid their experiences, they might be empowered by my narrative and eventually speak out. I saw their potential action in the same way that mine was influenced by other female black intellectual activists, and I hoped that our collective stories would lead to some transformative social impact. I further hoped to engage particularly (more) white women academics to fight for racial equality in society and organizations. Therefore, I sat down, went through my diary notes, and reached out to Shoikat.*

## Analysis of Vignettes

### Emerging Scholar

The ‘Emerging Scholar’ foregrounds a deceiving dimension of noncooperative spaces which theoretically appears to empower (aspirant) black female scholars by admitting them into predominantly ‘white-male’ dominated business schools, leading the individuals to believe in their potential impact, and the possibility of achieving a more equitable academia. Notwithstanding, noncooperative spaces are designed to disempower individuals such as Alice as soon as they enter academia. This disempowerment takes place primarily through underrepresentation and lack of any (substantial) support. Alice demonstrates this as a single black female scholar not only within her school but, apparently, countrywide. She is allowed into a system that perpetuates the tokenization of her body as representing all black women and black people (Bell, 1990). Essentially, through the underrepresentation (marginalization and isolation) of ‘Alice’, noncooperative spaces effectively obstruct the formation of a critical mass internally to disrupt ‘old white traditions’ (Lagermann, 2013). Thus, while noncooperative spaces do not overtly prohibit intellectual activism (as illustrated by Alice’s successful collaboration), their admission of one single black female scholar caters to the preservation of white elites’ interests and white patriarchal power (Liu, 2019a).

### White Power and Disruption

In this vignette we observe an interconnection between the two aspects of noncooperative spaces—namely, *false equivalence* and a *deceiving design*. *First*, a white female individual such as Lina applies false equivalence to racism, corruption and gender inequality, leading her to qualify the effects of the last two dimensions homogenously with the dehumanization of black bodies based on skin pigment (racism). *Second*, noncooperative spaces breed white power (Liu & Pechenkina, 2016), which can be destructive when threatened. White power, as a set of ideologies and an ordering principle that defines “the meaning and movements of bodies [and] subjectivities”, is displayed by white individuals such as Lina through dominance and distinction (King et al., 2007). An actor like Lina exercises her white power in how she redefines and transforms a home occupied by a black woman such as Alice into a noncooperative space for that non-white individual.

Lina achieves that transformation by moving in with Alice and appearing to embrace black women and sharing spaces with such individuals and, subsequently, by setting rules and parameters that do not seem explicitly racist. This essentialized notion of difference and dominance reified in noncooperative spaces implies that someone like Lina not only controls a black woman such as Alice, but also deploys corporal punishment to re-articulate and restore her white power when her rules are disobeyed by the latter (Alice). This manifests against a backdrop of multiple noncooperative spaces that implicitly instruct individuals like Lina that anti-racism statements issued and circulated in public or internally by organizations and institutions are immaterial to white power, as exemplified by the blue-chip company’s actions. Thus, racist behavior (by individuals like Lina) is not sanctioned within, and across, both organizations and institutions, insofar as powerful white actors remain indifferent to racism, with the only distinction being organizational/institutional abstinence from explicitly racist discourses (Ansell, 2016).

Within such diverse organizational, institutional and social contexts, official rejection of racism operates in parallel to internal practices and structures that effectively embed racism and reify white power. Another illustrative embodiment of such noncooperative spaces is the (Western) police institution that traditionally devalues women by showing them that they do not belong within it (Prokos & Padavic, 2002). Therefore, the (mostly white) women joining the (Western) police system are inclined to assimilate into its male hegemonic structures (Prokos & Padavic, 2002) and emulate the ‘boys’ to attain legitimacy (Horn, 1997).

Historically, the same (Western) police system is essentially indifferent to racism and may (overtly or otherwise) promote racist violence (Durán & Shroulote-Durán, 2021). Indifference is particularly observable in the (assimilated) two white female police officers’ friendly treatment of Lina and refusal to name racism for what it is, or treat such violence with urgency. Experiences acquire meaning based on how they are named by powerful (white) actors (Bandura, 1999). Hence the female police officers’ loose and instant categorization of a racially motivated physical assault as domestic violence obscures the act as racism and reflects an indifference to racist practices committed by white female individuals such as Lina. Effectively, this halts any further legal interventions targeting racial equality.

### Let Down

The relevance of the vignette ‘Let Down’ lies in exposing the link between powerful and presumably pro-racial equality white actors’ indifference to racism, and their subsequent abandonment of victimized black female individuals such as Alice when they seek recourse. Abandonment within a noncooperative space resembles powerful white actors’ complete abdication of obligations and duties towards a racialized (and any marginalized) individual, despite the agents’ exclusive position as recourse (Salerno, 2012) and open invitation to the victims to seek support. Thus, black female scholars must fight for themselves in order to survive within noncooperative spaces.

To exemplify, the female police officers’ inaction towards and trivialization of the potential damage of the attack epitomizes police indifference to racism that deliberately forgets and deserts victims like Alice, and ignores racist perpetrators. Similarly, the indifference mirrored in the landlord’s failure to provide Alice with safe accommodation embodies an abdication of responsibilities that adds layers of victimization upon racialized individuals such as Alice. This victimization is reflected by how Alice must incur additional accommodation costs to continue to work safely and keep her temporary job during the COVID-19 pandemic.

Therefore, noncooperative spaces—such as the police force, housing system, business schools, and a social context such as Alice’s ‘redefined home’—are not visibly linked and coordinated in their daily operations. Nevertheless, they often collude covertly in victimizing racialized bodies, and totally disregard their obligations with catastrophic consequences for the targeted individuals (Salerno, 2012), despite endorsing racial equality.

### Falling Star

The vignette ‘Falling Star’ surfaces how indifference to racism among powerful white actors in noncooperative spaces indirectly fosters and generates negative experiences that potentially bring down an (aspirant) black female intellectual activist. We observe this in Alice’s struggle with mental balance and subsequent hiding of the experience, her anger and humiliation, perception of hegemonic actors’ suspicions around her motives should she reveal the assault, gradual withdrawal from public engagement with intellectual activism, and questioning her work’s worth. Alice’s struggle with mental stability stems from having to conceal a traumatic racist experience from her white family, white friends, and predominantly white students and workmates in a noncooperative space. Experience shows that (powerful) white actors link black female scholars’ work to self-interest rather than regarding it as a genuine effort to establish a more equitable and humane society (Bell & Nkomo, 1999). Thus, fear of being judged—and potentially raising suspicion even among family members—progressively silences and alienates individuals such as Alice (cf. Lundberg-Love et al., 2011).

Furthermore, to the extent that a physical assault is a humiliating and degrading experience for anyone, abandoned and silenced (black) victims such as Alice tend to negatively evaluate themselves, question their worth, and become discouraged (Lundberg-Love et al., 2011). Such acts of self-devaluation subsequently manipulate individuals into attacking their work, its purpose, and its worth. This progresses to self-doubt (Frost et al., 1979), specifically around the black female scholar’s competencies for intellectual activism and potential contribution towards achieving racial equality (Braslow et al., 2012). Hence, noncooperative spaces seduce (aspirant) intellectual activists to fight for racial equality (Liu, 2019a); however, such spaces can also insidiously destroy individuals as exemplified by Alice’s experience.

### Re-emerging

The final vignette, ‘Re-emerging’, points to the significance of critical reflexivity and courage in empowering (aspirant) black female scholars to re-gain their lost voices within noncooperative spaces. Critical reflexivity involves interrogating assumptions, values and experiences underlying a black female scholar’s engagement and its (perceived) impact (Cunliffe, 2004)—this process precedes the writing up of an autoethnography and, thus, should not be conflated with the self-reflexivity that informs the construction of the narrative itself (Ellis et al., 2011). Alice’s sensemaking revolves around questioning who she is, and how she relates to role model black female scholars’ work, sacrifices, and the potential risks they take, as well as the world around her, as a way for her to form a bigger picture and serve a larger purpose. She also critically reflects on her past actions and possible futures to inform her decision on speaking out (cf. Jun 1994, cited in Cunliffe, 2004).

Initially Alice’s engagement with intellectual activism is driven by the absence of black/African social realities from business education and research. Nonetheless, as Alice stumbles along the journey, she draws courage from other black female scholars that she regards as role models. Contrary to bravery that implies boldness and determination, or an ability to fearlessly and often intuitively confront danger or pain (Kinsella et al., 2017), courage is not the absence of fear. Rather, courage involves acting deliberately following (critical) reflection to pursue a collectively valued moral goal in the presence of perceived risks, threats, and obstacles (Goud, 2005; Koerner, 2014; Rate & Sternberg, 2007).

Black scholars’ courageous acts help and support other marginalized individuals who perceive such actions (Worline & Quinn, 2003). Thus, Alice emulates those scholars and demonstrates courage by disclosing her “unspeakable” experience (van de Berg, 2021). Revealing her story to the public invites “stigmatizing and negative consequences for healing” (van de Berg, 2021), and dangerous repercussions for her career from predominantly white (male) leadership within noncooperative spaces. Yet Alice courageously chooses to speak out. Her courageous action further contradicts traditional assumptions traced back to early Western philosophers such as Plato and Aristotle (Rate & Sternberg, 2007), that attribute courage exclusively to white men belonging to the upper social class (Jablin, 2006).

Alice’s action also challenges contemporary scholarship that portrays courage as embodying stable properties (Hanna et al., 2021) intrinsic in some individuals and not others (Solinger et al., 2020). Courage is not exclusively inherent in some (white) male individuals as it can be acquired by (aspirant) black female activists like Alice. Furthermore, far from being stable, courage is fragile, transient, and prone to depletion; yet it can regenerate and be replenished. Therefore, we suggest that courage represents a resource that is accumulated, and sometimes lost by an individual depending on what Solinger et al. (2020) describe as ‘triggers within the environment’. Alice loses her courage following the physical assault. Her courage is, however, revived by multiple inconsistent events.

*First*, there are more threats to racial equality globally as reflected in the murders of black people by police, and the disproportionate COVID-19-related deaths among ethnic minorities. *Second*, and in parallel, more voices are campaigning for racial equality thus pressuring (powerful) institutions and organizations to reflect on their agency and admit their failure to tackle racism (Logan, 2019). These dimensions, coupled with Alice’s critical reflection, converge to replenish and trigger her courage.

Notwithstanding, when aspirant intellectual activists such as Alice lose courage, their voice vanishes too. In essence, having courage (to speak out) in a noncooperative space means that black female intellectual activists may stumble and fall—i.e., (temporarily) lose courage and their voice. However, their extraordinary commitment to racial equality and the progress of their black social group gives them a higher purpose, allowing them to re-emerge or at least attempt such comeback (Kinsella et al., 2017). Nevertheless, possessing courage does not exempt a victimized individual such as Alice from feeling humiliated as implied in her narration of being beaten up by a white woman whose qualifications and income are apparently beneath hers. Although humiliation can be a powerful tool wielded by white actors to intimidate black individuals who fight for racial equality, it does not permanently curtail courage, voice, and the struggle for racial equality.

## Discussion

### Courage and Intellectual Activism in Noncooperative Spaces

Individuals such as Alice continuously encounter racially motivated domestic violence and work experiences due to a confluence of indifference to racism, false equivalence of racism with other structural inequalities, and feigned support for racial equality by (some) seemingly disaggregated powerful white actors across contexts. As Alice’s vignette ‘Let Down’ illustrates, the Western judicial system, housing estate system, and a racist academia are separate entities that publicly endorse racial equality, yet simultaneously conspire and collude invisibly to establish noncooperative spaces. While business schools collude through a disempowering underrepresentation and alienation of black female scholars, the (Western) housing and police systems conspire through indifference to racism that intensifies the suffering of racialized bodies, as exemplified by Alice’s journey.

The collusion of powerful white actors in noncooperative spaces only becomes visible when individuals such as Alice can tell their story. As history testifies, black female intellectuals do not resign themselves to the constraints of white power (Gore, 2011). Rather, they courageously continue speaking truth to power and telling truth to people (Collins, 2013) by exposing and challenging racism to stop its harm, despite recurring threats to black bodies’ (professional) existence. However, the status of black female intellectual activists as marginalized stakeholders (Derry, 2012) limits their access to resources for transforming noncooperative spaces and achieving racial equality. Sustainable efforts and transformation thus demand the contribution of particularly ‘good [white] people’ (Rate & Sternberg, 2007) —*white allies*—due to their relatively more empowered social location as compared to that of any other social group (Bell et al., 2021; Contu, 2020; Edmondson et al., 2020). By ‘good people’ (Rate & Sternberg, 2007), we mean white individuals who aspire to establish a more equitable academia and world, and who have the resources to align themselves with such a goal (Dean, 2019).

### Theoretical Implications

#### Agential Level

Researchers assert that, “when ‘good [white] people do nothing (‘i.e., when they fail to act when the situation necessitates an *appropriate* action”, Rate & Sternberg, 2007, p. 4), they allow noncooperative spaces to continue breeding racism and embedding other structural inequalities within institutions, organizations, and societies. While a few white scholars are now more visibly speaking out against racism (e.g., Contu, 2020; Grimes, 2001; Swan, 2017), the prevalence and continuation of noncooperative spaces suggests that still not enough ‘good [white] people’ are committed to racial equality despite their desire for all forms of equality. We realize that challenging powerful white actors’ unresponsiveness to racism and inclination to feign support for black female intellectual activists and racial equality are all oppositional to hegemonic norms and expectations underpinning noncooperative spaces (Collins, 2013; Crenshaw, 1991).

Thus, ‘good’ white people (allies) may face and fear repercussions for their activism. To manage fear, such agents ought to develop courage individually and, more fundamentally, as members of a collective who support one another and collaborate to combat racism. We ground this idea on scholarship around *courageous collective actions* (Quinn & Worline, 2008; Worline & Quinn, 2003), which we interpret “as constructive confrontation” performed by white allies in cooperation with black and brown individuals, with the aim to dismantle the racist and deeply marginalizing status quo of a noncooperative space (Quinn & Worline, 2008, p. 498)

Courageous collective action is predicated on a moral dimension that speaks to an individual’s inner standards, and propels the (white) individual to undertake good actions for the sake of others irrespective of potential threats to the self (Sekerka et al., 2009). To effectively partake in courageous collective action and achieve racial equality, white allies must be willing to overcome their “shame and humiliation in order to admit” and reject false equivalence, indifference to racism, and the deceiving features underpinning noncooperative spaces (Miller, 2000). Another theoretical precondition for courageous collective action pertains to white allies’ acceptance of black (and brown) female scholars as legitimate leaders within organizational, institutional and social contexts, instead of viewing these non-white bodies with suspicion (Mayo & Morgan Roberts, 2019). As racialized bodies, black (and brown) individuals can better articulate their (racialized) social realities, and actively inform—as well as implement—strategies for eliminating racism (Chowdhury, 2021b).

In addition, courageous collective action for racial equality demands that white allies engage in creating and implementing radical plans (Contu, 2020) jointly with black (and brown) individuals to demolish noncooperative spaces across contexts. As Tuomela (2013) metaphorically exemplifies: When two individuals paint a house, A might paint the front and B might paint the back. Thus, they collaborate to achieve a common goal. In much the same way, it follows that “there must be jointness or togetherness” (Tuomela, 2013, p. 11) in demolishing noncooperative spaces and achieving racial equality.

Beyond these above-stated suggestions, we highlight the urgency to co-create narratives (Quinn & Worline, 2008; Worline & Quinn, 2003) that unite white allies and black and brown individuals, rather than foster a divide between ‘us (white individuals) versus them (non-white individuals)’. Narratives created and shared by diverse individuals build collective identities and guide action when ‘good’ white scholars work jointly with black and brown scholars (Quinn & Worline, 2008) to eliminate racism. Narratives not only ascribe meaning to a collective struggle but also help build individuals’ courage within noncooperative spaces, ultimately fueling courageous collective action (Quinn & Worline, 2008).

#### Organizational and Institutional Level

To “destabilize” and “obliterate” noncooperative spaces (Ballard et al., 2020, p. 592), agents have to cultivate courageous collective action across contexts on a much larger scale (Le Pennec & Raufflet, 2018; Roberts & Bradley, 2005). Hence, we draw inspiration from studies on joint work by multiple institutions/organizations and actors within and across sectors, targeting a common goal (Diani & Ivano, 2004; Laasch et al., 2020; Le Pennec & Raufflet, 2018; Roberts & Bradley, 2005), to propose the idea of *cross-contextual cooperation* for racial equality. We define this as: ‘Cooperation to eliminate racism and achieve racial equality by institutions and organizations within the same, and across sectors, that are formally networked based on voluntary membership, mutual interest, and a common purpose*.*’ Theoretically, cross-contextual cooperation for racial equality involves diverse actors from the private, public, civil, and non-governmental sectors (including religious entities), who voluntarily ‘act together’ and ‘act collectively’ (Tuomela, 2013) to establish a more equitable society.

*Cross-contextual cooperation* demands a formal mandate and coordination, ideally within a global non-governmental entity such as the United Nations Principles of Responsible Management Education (cf. Laasch et al., 2020), in order to attain global legitimacy, better inclusion, and greater access to resources. Most fundamentally, various actors within cross-contextual cooperation for racial equality potentially effect change by pooling and coordinating their resources, and speaking out with one voice against noncooperative spaces and racism (Diani & Ivano, 2004; Laasch et al., 2020; Roberts & Bradley, 2005). This contrasts with the limited effects of ad hoc independent activities in pursuit of a shared goal (Roberts & Bradley, 2005).

Furthermore, cross-contextual cooperation for racial equality will support institutions/organizations and individuals to learn from one another and adopt best practices (Knoben & Oerlemans, 2006; Laasch et al., 2020; Le Pennec & Raufflet, 2018; Roberts & Bradley, 2005). As Swan (2017) observes, several white leaders do not know how to tackle racism. Such actors potentially learn from diverse corporate representatives/practitioners, researchers, policymakers, religious leaders/representatives, and intellectual and civil rights activists, among other stakeholders. These various (courageous) actors contribute and combine their knowledge, skills and competencies in transformative ways not achievable by a single institution/organization or individual in pursuit of racial equality.

Nevertheless, possible obstacles may emerge for cross-contextual cooperation for racial equality, such as lack of willingness among powerful actors to mobilize resources in novel ways towards eliminating racism (Logan, 2019). Other impediments may manifest as conflicting goals, approaches and values attached to the initiative by diverse actors (Huxham & Vangen, 2000) with different behavioral norms and standards and in multiple geographical locations (Knoben & Oerlemans, 2006). Furthermore, power and control (Oliver & Ebers, 1998) may impede joint anti-racism work. Even then, cross-contextual cooperation for racial equality has the capacity to globally convey the scale and seriousness with which institutions, organizations and social actors truly pursue racial equality.

## Conclusion

We have sought to contribute to Black Scholarship on intellectual activism and racial equality by employing the concept of noncooperative spaces. In utilizing this concept (noncooperative spaces) we have made an attempt towards offering a theoretical perspective that may help explain the persistent entrenchment of racism within organizational, institutional and social settings, despite powerful white actors’ endorsement of racial equality. Drawing on our lead author’s autoethnography of her journey as an aspirant intellectual activist, we have underscored courage as pivotal in enabling black female scholars’ ‘voice’ to be heard within noncooperative spaces. We have further proposed courageous collective action by agents who must speak with one voice across different institutional, organizational, and social contexts to achieve racial equality. While our awareness of the subjective nature of autoethnographic inquiry cautions us against generalizations, evidence abounds on the commonality of racialized experiences endured by black intellectual activists within Western-centric contexts (e.g., Dei, 2018; Rollock, 2019; Settles et al., 2021). We therefore hope that our work invites business ethics and MOS scholars to engage in honest debate about the intersecting racialized (and marginalizing) effects of noncooperative spaces for non-white individuals (and other marginalized individuals), and consequently propose tangible solutions targeting racial and all forms of equality within institutions, organizations and wider societies.
